# Phylogeny of the Diploid Species of *Rubus* (Rosaceae)

**DOI:** 10.3390/genes14061152

**Published:** 2023-05-25

**Authors:** Xin-Fen Gao, Xian-Hua Xiong, David E. Boufford, Yun-Dong Gao, Bo Xu, Cheng Zhang

**Affiliations:** 1 CAS Key Laboratory of Mountain Ecological Restoration and Bioresource Utilization, Chengdu Institute of Biology, Chinese Academy of Sciences, Chengdu 610041, Chinaxubo@cib.ac.cn (B.X.);; 2 College of Life Science and Biotechnology, Mianyang Teachers’ College, Mianyang 621000, China; 3 Harvard University Herbaria, Harvard University, 22 Divinity Avenue, Cambridge, MA 02138, USA

**Keywords:** diploid species, phylogeny, Rosaceae, *Rubus*, taxonomy

## Abstract

*Rubus* L. (Rosaceae, Rosoideae) contains around 700 species distributed on all continents except Antarctica, with the highest species diversity in temperate to subtropical regions of the northern hemisphere. The taxonomy of *Rubus* is challenging due to the frequency of polyploidy, hybridization and apomixis. Previous studies mostly sampled sparsely and used limited DNA sequence data. The evolutionary relationships between infrageneric taxa, therefore, remain to be further clarified. In the present study, genotyping by sequencing (GBS) reduced-representation genome sequencing data from 186 accessions representing 65 species, 1 subspecies and 17 varieties of *Rubus*, with emphasis on diploid species, were used to infer a phylogeny using maximum likelihood and maximum parsimony methods. The major results were as follows: (1) we confirmed or reconfirmed the polyphyly or paraphyly of some traditionally circumscribed subgenera, sections and subsections; (2) 19 well-supported clades, which differed from one another on molecular, morphological and geographical grounds, were identified for the species sampled; (3) characteristics such as plants with dense bristles or not, leaves leathery or papyraceous, number of carpels, instead of inflorescences paniculate or not, aggregate fruits and leaves abaxially tomentose or not, may be of some use in classifying taxa whose drupelets are united into a thimble-shaped aggregate fruit that falls in its entirety from the dry receptacle; and (4) a preliminary classification scheme of diploid species of *Rubus* is proposed based on our results combined with those from previous phylogenetic analyses.

## 1. Introduction

*Rubus* L. (Rosaceae; Rosoideae), a species-rich and economically important genus, is characterized by shrubs, subshrubs and herbs bearing stipules, usually prickles or bristles, and a compound fruit comprising an aggregation of drupelets. Species of *Rubus* are on all continents except Antarctica [1,2]. Except for a fraction of the species in the southern hemisphere, most species of *Rubus* occur in temperate to subtropical regions of the northern hemisphere, especially in temperate Eurasia and North America [3,4,5,6,7]. The exact species number of *Rubus* is unclear. The estimated number has ranged from ca. 200 species [8], ca. 437 species [9,10,11], 250–700 species [2], ca. 700 species [4,6,7] and 600–1000 species [12] to 900–1000 species [13]. Various species of *Rubus* are economically significant as fruit crops, ornamentals, invasive weeds and as pioneers in early forest succession [1,14].

The taxonomy of *Rubus* has historically been difficult due to morphological diversity and overlapping characteristics, apomixis, polyploidization and hybridization [1,5,15,16,17,18,19,20,21]. Morphologically, the species of *Rubus* exhibit tremendous diversity, with plants ranging from woody to semi-woody, trailing, erect or climbing shrubs or subshrubs to perennial creeping dwarf herbs. The leaves range from simple to palmately or pinnately compound with 3–11(–15) leaflets [1,7,15,22]. *Rubus* has a basic chromosome number of seven, and ploidy levels ranging from diploid to tetradecaploid (or octadecaploid) [5,13,20,23,24,25,26,27,28,29,30,31,32,33,34,35,36,37,38,39,40,41,42]. Hybridization in *Rubus* occurs mostly between closely related species, but also in some cases between species classified in different subgenera [1].

The most recent and the only global taxonomic treatment of *Rubus* was made by Focke [9,10,11] based on morphological characteristics. Focke divided *Rubus* into 12 subgenera: *Anoplobatus* (Focke) Focke, *Chamaebatus* (Focke) Focke, *Chamaemorus* (Hill) Focke, *Comaropsis* (Rich. ex Nestl.) Focke, *Cylactis* (Raf.) Focke, *Dalibarda* (L.) Focke, *Dalibardastrum* Focke, *Idaeobatus* (Focke) Focke, *Lampobatus* (Focke) Focke, *Malachobatus* (Focke) Focke, *Orobatus* (Focke) Focke and *Rubus* (subg. *Eubatus* Focke). Two subgenera, *Diemenicus* A. R. Bean (including only *Rubus gunnianus*) [43] and *Micranthobatus* (Firtsch) Kalkm. [44], were subsequently described. Among them, the three largest subgenera are *Idaeobatus*, *Malachobatus* and *Rubus*, the first two occurring mainly in Asia (especially in China); subg. *Rubus* is in Europe and North America [5]. The raspberries, one of the economically important crops of *Rubus*, mainly belong to subg. *Idaeobatus*, while the blackberries are primarily included in subg. *Rubus*. The subgenera *Anoplobatus*, *Dalibarda* and *Idaeobatus* are predominantly diploid, whereas subgenera *Dalibardastrum*, *Malachobatus* and *Orobatus* are almost entirely polyploid, and subg. *Chamaebatus*, subg. *Cylactis* and subg. *Rubus* contain a small number of diploid members [13,32].

Molecular data have shown *Rubus* to be sister to the clade comprising the genus *Rosa* L., tribe Sanguisorbeae DC., tribe Potentilleae Sweet and tribe Colurieae Rydb. [45]. Further studies found *Rubus* to be monophyletic, whereas among the long-recognized subgeneric taxa, except for subg. *Orobatus*, all other subgenera were paraphyletic or polyphyletic [1,42,46]. Previously, although a diversity of molecular markers, including *GBSSI-1*, *GBSSI-2*, *ITS*, *LEAFY*, *ndhF*, *PEPC*, *rbcL*, *rpl16*, *rpl20*-*rps12*, *rpl32-ndhF*, *trnK*, *trnL-trnF*, *trnS-trnG* and *trnV-ndhC* [1,14,21,37,46,47,48,49,50,51,52,53,54,55], were used to infer the phylogeny of *Rubus*, most of those studies applied to a limited number of species and molecular loci or were restricted to taxa within administrative boundaries, resulting in low phylogenetic resolution and sometimes even giving inconsistent results. The evolutionary relationships between the species of *Rubus* are in need of further clarification.

The rapid development of next-generation sequencing enables researchers to use genome-wide polymorphisms from different species for phylogenetic analysis. Reduced representation sequencing methods, such as genotyping by sequencing (GBS), can provide thousands of single nucleotide polymorphisms (SNPs) and have been used for phylogenetic analyses of both closely and distantly related taxa [56,57,58,59,60,61,62,63,64,65].

Given that the polyploid species of *Rubus* have a complex evolutionary history, this paper focuses on the phylogeny of diploid species of *Rubus*. We inferred a molecular phylogeny of diploid species of *Rubus* with thousands of SNPs identified by the GBS approach to (1) vigorously test the monophyly of subgenera, sections and subsections of *Rubus* based on larger sampling; (2) identify major clades within diploid species of *Rubus*; and (3) build the molecular foundation for future taxonomic revision of *Rubus*.

## 2. Materials and Methods

### 2.1. Taxon Sampling

The taxa were chosen to include as many diploids species of *Rubus* as possible. In total, 186 accessions, representing 65 species, 1 subspecies and 17 varieties of *Rubus*, constituted our ingroup. Except for subg. *Chamaebatus*, our sampling covered all subgenera containing diploid species and the major geographical range of the diploid species of the genus. Diploid representatives of *Fragaria* L. (one accession representing one species, *Fragaria daltoniana*) and *Rosa* L. (three accessions representing two species, *Rosa cymosa* and *Rosa luciae*) were selected as outgroups based on previous findings that *Rosa*, *Rubus* and three tribes (Sanguisorbeae, Potentilleae (including *Fragaria*) and Colurieae) constitute the super tribe Rosodae in the subfamily Rosoideae of Rosaceae. Voucher information for the sampled taxon are provided in the Appendix A.

### 2.2. Genotyping by sequencing (GBS) Dataset

Total genomic DNA was extracted from silica-dried material using the CTAB method [66]. The DNA was quantified, and its quality was evaluated using the following methods: (a) Agarose gel electrophoresis to test DNA purity and integrity, (b) Nanodrop spectrophotometer to test DNA purity (OD260/OD280), (c) Qubit Fluorometer to measure DNA concentration.

A reduced representation GBS library was prepared as described by Elshire et al. [56], using the restriction enzymes MseI and HaeII, and sequenced (paired-end reads) using the Illumina HiSeq platform at Novogene Bioinformatics Technology Co., Ltd., Beijing, China.

The raw data (raw reads) were first processed by fastp version 0.23.1 [67] in a series of quality control (QC) procedures using standards as follows: (a) removal of reads containing adapters; (b) removal of reads with >50% bases having phred quality < 5; and (c) removal of reads with ≥10% unidentified nucleotides (N). The remaining high quality clean reads were mapped to the reference genome (chloroplast genome, *Rubus coreanus* [68]; whole genome, *Rubus occidentalis* [69]) using the software BWA version 0.7.8 [70] with the command “mem -t 4 -k 32 -M”. In order to reduce mismatch generated by PCR amplification before sequencing, duplicated reads were removed by the help of SAMtools version 1.3.1 [71]. After alignment, we performed SNP calling on a population scale using a Bayesian approach as implemented in the SAMtools package, and then genotype likelihoods from reads for each individual at each genomic location and the allele frequencies in the sample were calculated with a Bayesian approach. The “mpileup” command was used to identify SNPs with the parameters as “-q 1 -C 50 -t SP -t DP -m 2 -F 0.002”. The resulting SNPs were filtered by script with the parameters “dp 4, miss 0.5 and maf 0.01′” to obtain high quality SNPs for subsequent phylogenetic analysis.

### 2.3. Phylogenetic Analysis

The chloroplast and nuclear datasets were analyzed separately. Equally weighted maximum parsimony (MP) jackknife (JK) analyses [72] were conducted using 1000 random-taxon-addition replicates and tree-bisection-reconnection (TBR) branch swapping in PAUP* version 4.0a169 [73], with MAXTREES set to 10,000 and the removal probability set to approximately 37%, and “jac” resampling emulated analyses. Insertions and deletions were coded as missing data. Jackknife 50% majority-rule consensus trees were computed.

jModelTest 2 [74,75] was used to test the models of nucleotide substitution for maximum likelihood (ML) [76]. The Akaike information criterion (AIC) [77] was used to select among models instead of the hierarchical likelihood ratio test (hLRT), following Pol [78] and Posada and Buckley [79].

Maximum Likelihood (ML) analyses were performed using RAxML-HPC2 on XSEDE version 8.2.12 [80] on the CIPRES web server [81], with 1000 rapid bootstrap analyses followed by a search for the best-scoring tree in a single run [82]. The nucleotide substitution models “GTRCAT” and “GTRGAMMA” were chosen for the bootstrapping phase for the chloroplast dataset and nuclear dataset, respectively.

## 3. Results

### 3.1. GBS Data Analysis Summary

A summary of the sequence data generated from the samples studied is included in Supplementary Material Tables S1–S3. The 190 accessions, which consisted of 186 samples of *Rubus* and 4 samples of outgroups, were successfully sequenced with the Illumina HiSeq sequencing platform. The mean GC content was 37.85%, which is within the normal range. The sequencing data were high-quality (Q20 ≥ 96.19%, Q30 ≥ 89.54%), and subsequent analyses could be performed. After excluding the outgroups, the average sequencing depth of the *Rubus* samples ranged from 10.81 to 75.48, with an average of 25.21. The average degree of sequence coverage from the samples of *Rubus* was 14.51% for at least single-base coverage, while the average degree of coverage of the samples with at least 4-base coverage was 8.68%. A total of 7,293,296 SNPs (nuclear genome) and 8115 SNPs (chloroplast genome) were obtained according to detection with SAMtools software; 256,954 high-quality SNPs (nuclear genome) and 1848 high-quality SNPs (chloroplast genome) were obtained after filtration.

### 3.2. Phylogenetic Tree Based on SNPs of Nuclear Genome

**Figure 1:**
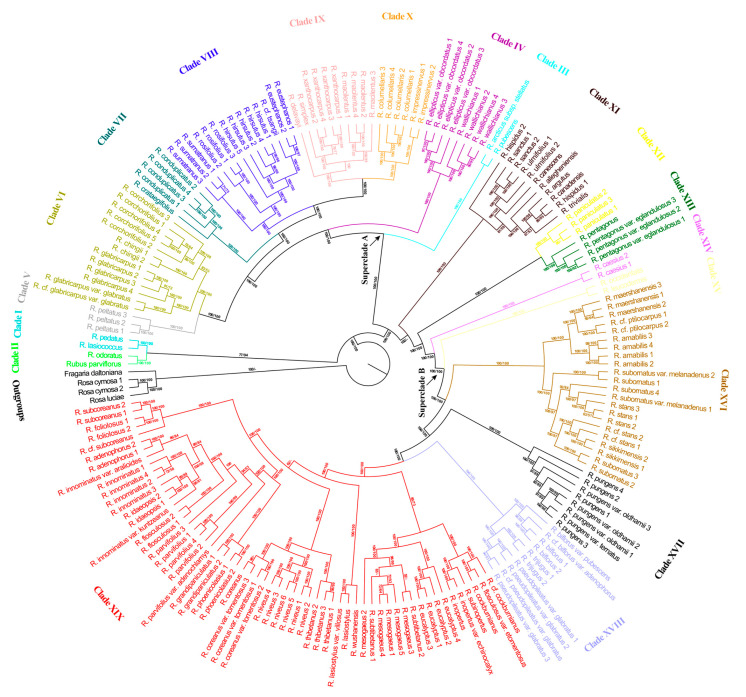
Maximum likelihood phylogeny of *Rubus* based on SNPs of nuclear genome. Maximum likelihood bootstrap support values (ML BS) and maximum parsimony jackknife support values (MP JK) are shown along the branches (symbol “-” indicates that the relationship or clade is not supported by MP analysis).

Both maximum likelihood (ML) phylogeny and maximum parsimony (MP) jackknife (JK) analyses based on high-quality SNPs from the nuclear genome showed *Rubus* to be monophyletic (Figure 1).

ML bootstrap (BS) support and MP JK support are shown on the phylogenetic tree from the ML analysis. Based on our reconstructed nuclear phylogeny and in consideration of morphological characteristics and distribution information, 19 maximumly supported (ML BS = 100%, MP JK = 100%) clades of *Rubus* were identified in this study (Figure 1). The relationships among most of the 19 clades were also well supported.

The topologies and support values from ML and MP analyses were mostly similar except for the position of Clade XI. The ML analyses resolved Clade XI as sister to the lineage containing Clade XII–Clade XIX with maximum support values. In contrast, the MP analysis resolved Clade XI as sister to the remaining clades except for Clade I and Clade II; support values were also maximum.

Clade I plus Clade II were resolved as monophyletic with 77% ML BS and 94% MP JK support and as sister to the rest of the ingroup.

Except for Clade XI, the basal Clade I plus Clade II, and the predominately polyploid Clade XII, Clade XIII and Clade XIV, the remaining clades formed two strongly supported superclades. Superclade A contained Clade III–Clade X, and Superclade B consisted of Clade XV–Clade XIX.

### 3.3. Phylogenetic Tree Based on SNPs of Chloroplast Genome

**Figure 2:**
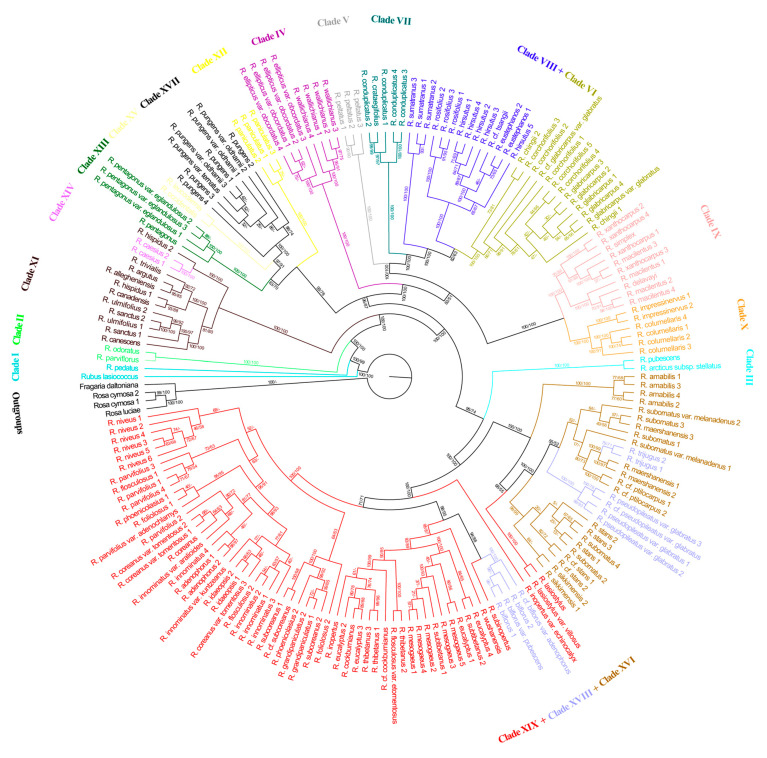
Maximum likelihood phylogeny of *Rubus* based on SNPs of chloroplast genome. Maximum likelihood bootstrap support values (ML BS) and maximum parsimony jackknife support values (MP JK) are shown along the branches (symbol “-” indicates that the relationship or clade is not supported by MP analysis).

The topologies from the ML and MP analyses were similar for the chloroplast phylogeny, which also showed *Rubus* to be a monophyletic group. However, the topologies and support values based on the chloroplast data were not identical to those based on the nuclear data (Figure 2). First, for the topology of the chloroplast tree, 13 (Clades II, III, IV, V, VI, VII, IX, X, XII, XIII, XIV, XV and XVII) of the 19 major clades identified from the nuclear data were reconfirmed, but the monophyly of 6 clades (Clades I, VIII, XI, XVI, XVIII and XIX) was not supported. *Rubus lasiococcus* did not cluster with *Rubus pedatus*. The accessions of Clade VIII identified by nuclear data were split into two clusters on the chloroplast tree, one of which (including *Rubus eustephanos*, *Rubus hirsutus*, *Rubus rosifolius*, etc.) was sister to Clade VI. Clade XVI clustered with a portion of the accessions in Clade XVIII (*Rubus pseudopileatus* var. *glabratus*, *Rubus trijugus*, etc.) and *Rubus lasiostylus* and *R. lasiostylus* var. *villosus* of Clade XIX. Species in the remainder of Clade XIX clustered with some of the accessions in Clade XVIII (*Rubus biflorus*, etc.). Second, the two superclades were not supported by the chloroplast data. Third, Clade III was sister to the lineage containing Clades XVI, XVIII and XIX, instead of the lineage containing Clades IV to X. Clade XIV nested within Clade XI and was not sister to Superclade B as identified by nuclear data. Clade XVII was more closely related to Clade XV than to Clades XVIII and XIX.

## 4. Discussion

### 4.1. Phylogenetic Incongruence between the Chloroplast and Nuclear Phylogenies

The monophyly of *Rubus* in its current circumscription was maximally supported in this study (Figure 1 and Figure 2) and consistent with the results of earlier studies [1,42,46], further confirming *Rubus* to be a natural group. Phylogenetic incongruence, however, was detected between the chloroplast and nuclear phylogenies as the topologies based on the chloroplast data were not identical to those based on the nuclear data. The discordance between the nuclear and chloroplast phylogenetic relationships may be due to incomplete lineage sorting, genetic introgression and hybridization.

### 4.2. Major Evolutionary Lineages within Rubus Revealed in This Study

Within the ingroups, the 186 accessions representing 65 species, 1 subspecies and 17 varieties of *Rubus* included in the current study were assignable to the following 19 well-supported clades (Figure 1). Those clades are also supported by morphological characteristics.

The clades (Figure 1) can be divided into five main groups and thus may represent several major evolutionary lineages within *Rubus*, which is somewhat consistent with the results of Carter et al. [42]. The first group, found primarily in North America and containing Clades I and II, represents the basal lineage of *Rubus*. The second group was composed of Clade XI and mainly includes members of *Rubus* bearing aggregate fruit that falls together with the receptacle. The third group consists of Superclade A, including Clades III to X. The fourth group contains Clades XIII and XIV and represents the polyploid species of *Rubus*. The fifth group consists of Superclade B, including Clades XV to XIX. Unfortunately, morphological homoplasy is common in *Rubus*, thereby making it challenging to distinguish Superclades A and B from the other groups. That is, morphological synapomorphies of the two superclades are unclear at present.

Clade I––Clade I, composed of *R. lasiococcus* and *R. pedatus*, was shown to be sister to Clade II with high support (ML BS = 77%, MP JK = 94%; Figure 1). *R. lasiococcus* is native to North America and *R. pedatus* occurs naturally in North America and NE Asia. Both species were included in *R.* subg. *Dalibarda* by Focke [9,11]. They are morphologically similar in their creeping herbaceous habit, unarmed stems, broad stipules and filiform filaments.

Clade II––Clade II contained two species, *Rubus odoratus* and *Rubus parviflorus*, and was sister to Clade I (Figure 1). The sister relationship between *R. odoratus* and *R. parviflorus* was consistent with the findings by Carter et al. [42]. The two species, both native to North America and included in *R.* subg. *Anoplobatus* by Focke [9,11], are distinguished from other species of *Rubus* by habit (erect, prickleless shrubs) and their simple palmately lobed or divided leaves. Our study, together with previous studies [1,42], provides strong molecular evidence that *R.* subg. *Dalibarda*, *R.* subg. *Anoplobatus* and *R.* subg. *Chamaemorus* occupy basal positions in the phylogenetic trees, which conflicts with Lu’s [5] hypothesis of *R.* subg. *Idaeobatus* being the most primitive group.

Clade III––Clade III contained *Rubus arcticus* subsp. *stellatus* and *Rubus pubescens* and was strongly supported (ML BS = 100%, MP JK = 100%) as sister to the lineage containing Clades IV to X (Figure 1). Clade III to Clade X together form the lineage Superclade A, the morphological synapomorphy of which is unclear at present. *R. arcticus* subsp. *stellatus* occurs in North America and the Russian Far East; *R. pubescens* is in North America. Morphologically, they are somewhat similar to the members of Clade I, but differ from the latter in having dilated, laminar filaments.

Clade IV––Clade IV, composed of eigh samples of *Rubus ellipticus* var. *obcordatus* and *Rubus wallichianus*, was strongly supported (ML BS = 100%, MP JK = 100%) as sister to the clade containing Clades V to X (Figure 1 and Figure 2). The species of Clade IV are endemic to Asia [6,7] and were included in *R.* subg. *Idaeobatus* sect. *Idaeanthi* ser. *Elliptici sensu* Focke [10,11]. The morphological synapomorphies of Clade IV are shrubs with sparse, curved prickles and dense, spreading reddish brown bristles and usually three-foliolate leaves.

Clade V––This well-supported clade contained only one species, *Rubus peltatus* of China and Japan. Clade V was sister to Clade VI with maximum support (Figure 1). *R. peltatus* differs from other species of *Rubus* in having peltate leaves. Our molecular analysis supported the treatment of Focke [10,11], Yu and Lu [83], Lu and Yu [6] and Naruhashi [84], who placed *R. peltatus* in the monospecific section *R.* subg. *Idaeobatus* sect. *Peltati* (Lu and Yu’s section and subsection, respectively, almost corresponding to Focke’s subgenus and section).

Clade VI––In our samples, Clade VI contained *Rubus corchorifolius*, *Rubus chingii*, *Rubus glabricarpus* var. *glabricarpus* and *R. glabricarpus* var. *glabratus*. The sister relationship between Clade VI and Clade V was well supported (ML BS = 100%, MP JK = 100%) in our analyses (Figure 1). The four taxa of Clade VI are distributed in Asia and were included in *R.* sect. *Idaeobatus* subsect. *Corchorifolii* by Lu and Yu [6]. Morphologically, species of Clade VI share the following features: flowers usually solitary, leaves simple, stipules adnate to base of petiole, stems usually pilose or glandular (except in *R. chingii*), aggregate fruit nearly globose and hairy or glabrous. The members of this clade are similar in appearance to taxa included in Clade VII, with the main difference being that the latter have three to several flowers in nearly corymbiform inflorescences and glabrous aggregate fruit. Clade VI can be divided into two subclades. The first contains *R. corchorifolius* and *R. chingii*. Species of this subclade lack glandular hairs and the fruits are hairy. Members of the second subclade, represented by *R. glabricarpus* var. *glabricarpus* and *R. glabricarpus* var. *glabratus*, have glandular hairy pedicels and glabrous fruits.

Clade VII––Clade VII comprised two species—*Rubus crataegifolius* and *Rubus conduplicatus*—in our sampling and was strongly supported (ML BS = 100%, MP JK = 100%) as sister to a monophyletic lineage composed of Clades VIII, IX and X (Figure 1). Species of Clade VII are morphologically similar to each other in their nearly corymbiform inflorescences with three-to-several flowers, simple leaves, stipules adnate to the base of the petiole, aggregate fruit nearly globose and glabrous.

*R. chingii*, *R. conduplicatus* (*Rubus trianthus*), *R. corchorifolius*, *R. crataegifolius*, *R. glabricarpus*, *Rubus grayanus*, *Rubus microphyllus*, *Rubus palmatus* and *Rubus pseudoacer* were all included in *R.* subg. *Idaeobatus* sect. *Corchorifolii* by Focke [10,11], Yu and Lu [83] and Lu and Yu [6], a classification different from the taxonomic treatment of Naruhashi [84] and Nakai [85,86], who further segregated those species into a group with solitary flowers (*R.* subg. *Idaeobatus* sect. *Villosi sensu* Nakai and sect. *Corchorifolii sensu* Naruhashi) and a group with nearly corymbiform inflorescences (*R.* subg. *Idaeobatus* sect. *Crataegifolii sensu* Nakai and sect. *Microphylli sensu* Naruhashi). Our data (Figure 1 and Figure 2) suggested that *R. corchorifolius*, *R. chingii*, *R. glabricarpus* and *R. glabricarpus* var. *glabratus*, all bearing solitary flowers, form a monophyletic lineage (Clade VI). *R. crataegifolius* and *R. conduplicatus*, both bearing nearly corymbiform inflorescences, formed Clade VII. However, Clade VI + Clade VII were not shown to be monophyletic; that is, our results supported the above mentioned classification of Naruhashi [84] and Nakai [85,86], but not those of Focke [10,11], Yu and Lu [83] and Lu and Yu [6].

Clade VIII––In our sample, Clade VIII contained *R. eustephanos*, *R. hirsutus*, *R. rosifolius*, *Rubus sumatranus* and *Rubus* cf. *tsangii*, and was strongly supported (ML BS = 100%, MP JK = 100%) as sister to the monophyletic lineage formed by Clades IX and X (Figure 1). Species of Clade VIII are mainly in Asia. Morphologically, they share a shrubby habit, imparipinnate leaves and ca. 100 or more carpels usually inserted on a stipitate torus. Our molecular evidence supported the classification of Focke [10,11], Yu and Lu [83], Lu and Yu [6] and Naruhashi [84], who placed these species in *R.* subg. *Idaeobatus* sect. *Rosifolii* as a natural group.

Clade IX––Clade IX is composed of four endemic Asian species—*Rubus delavayi*, *Rubus macilentus*, *Rubus simplex* and *Rubus xanthocarpus*. The sister relationship between Clade IX and Clade X was well supported (ML BS = 100%, MP JK = 100%; Figure 1 and Figure 2). Morphologically, species of Clade IX are characterized by subshrubs or herbs’ leaves with three (or five) leaflets, and the abaxial surface of the calyx is pubescent and with straight needle-like or curved minute prickles (except in *R. macilentus* where the calyx is unarmed). *R. simplex* and *R. xanthocarpus* were both previously included in *R.* subg. *Cylactis* by Focke [9,11] for their suffruticose or nearly herbaceous habit. Yu and Lu [83], Lu and Yu [6] and Lu and Boufford [7], noting that the two species are morphologically similar to some members of *R.* subg. *Idaeobatus* in non-paniculate inflorescences and that the abaxial surface of the leaflets and aggregate fruit were not tomentose, transferred them to *R.* sect. *Idaeobatus sensu* Lu and Yu [6]. Our study (Figure 1 and Figure 2) showed that *R. simplex* and *R. xanthocarpus* are more closely related to *R. macilentus* of *R.* subg. *Idaeobatus* than to *R. pubescens* of *R.* subg. *Cylactis*, which supported the classification of Lu and Yu [6] and Lu and Boufford [7].

Clade X––Clade X consisted of four samples of *Rubus columellaris* and two samples of *Rubus impressinervus*. It was resolved as sister to the monophyletic Clade IX with maximum support (Figure 1 and Figure 2). The two species are morphologically quite different from the other species of *Rubus* that we sampled in having approximately leathery leaves. Our study suggests that *R.* sect. *Idaeobatus* subsect. *Leucanthi sensu* Lu and Yu (including *R. columellaris*, *R. delavayi*, *R. impressinervus*, etc.) [6] is paraphyletic and implies that the species with leathery leaves in *R.* subg. *Idaeobatus* may be a natural group and that *R. delavayi* might be excluded from *R.* subg. *Idaeobatus* sect. *Leucanthi.*

Clade XI––Clade XI was composed of *Rubus allegheniensis*, *Rubus argutus*, *Rubus canadensis*, etc. ML analyses of nuclear data indicated that this clade is strongly supported (BS = 100%) as sister to the monophyletic lineage formed by Clade XII–Clade XIX (Figure 1). The morphological characteristics of this clade include shrubs being often prickly, leaves usually ternate, pedately or palmately and quinately compound, persistent narrow stipules which are mostly adnate to the base of the petiole, drupelets remaining on the fleshy receptacle at maturity or falling with the receptacle, or falling separately. Geographically, the members of this clade are mainly American and Eurasian. The eight species of this clade and *Rubus caesius* (4*x*) of Clade XIV were all included in *R.* subg. *Eubatus* (= subg. *Rubus*) by Focke [11]. Although the nine species clustered together based on chloroplast data (Figure 2), the phylogenetic analyses based on our nuclear data (Figure 1), ITS [1] and nearly one thousand low copy nuclear genes [42] suggested that *R.* subg. *Rubus* is not monophyletic.

Clade XII––Clade XII contained three samples of *Rubus paniculatus*, one of the few doubtfully diploid species reported to occur in the predominately polyploid *R.* subg. *Malachobatus* [27]. This clade was strongly supported (ML BS = 100%, MP JK = 100%) as sister to Clade XIII based on the nuclear data (Figure 1). The remarkable morphological characteristics of Clade XII include shrubs that are often prickly, stems erect, arching or climbing and broad, and free stipules that are caducous or persistent on the twig near the base of the petiole.

Clade XIII––Clade XIII, comprising the Asian endemics *Rubus pentagonus* var. *pentagonus* (tetraploid [42]) and *R. pentagonus* var. *eglandulosus*, was sister to Clade XII with maximum support (Figure 1). Species of Clade XIII are morphologically distinguishable from other sampled taxa by their shrubby habit and palmately compound leaves with three or five leaflets. The close phylogenetic relationship between *R. pentagonus* of subg. *Idaeobatus* and *R. paniculatus* of subg. *Malachobatus* is consistent with the findings of Wang et al. [46] and Carter et al. [42] and may provide some support to the hypothesis that *R. pentagonus* may be one of the possible progenitors of the subg. *Malachobatus* polyploids [42,46].

Clade XIV––Clade XIV consisted of two samples of *R. caesius* (Figure 1), a tetraploid of subgenus *Rubus* occurring naturally from Europe and western Asia to western China. Our chloroplast phylogeny showed that *R. caesius* was nested within Clade XI (ML BS = 100%, MP JK = 100%; Figure 2), indicating that the maternal parent of *R. caesius* was likely from *R.* subg. *Rubus*, which is consistent with the findings of Carter et al. [42].

Clade XV––Clade XV contained two North American species, *Rubus leucodermis* and *R. occidentalis*. The nuclear phylogeny indicated that Clade XV was sister to the lineage formed by Clades XVI through XIX (ML BS = 100%, MP JK = 100%; Figure 1). Clade XV to Clade XIX together form superclade B (ML BS = 100%, MP JK = 100%; Figure 1), the morphological synapomorphy of which is unclear at present. Morphologically, the two species of Clade XV share palmately compound or ternate leaves, nearly black aggregate fruit and drupelets separating from the torus. *R. leucodermis*, *R. occidentalis* and two species, *Rubus eriocarpus* and *Rubus glaucus*, which were not sampled, were all placed in *R.* subg. *Idaeobatus* sect. *Idaeanthi* ser. *Occidentales* by Focke [9,11]. In the molecular phylogeny the three diploid species, *R. leucodermis*, *R. occidentalis* and *R. eriocarpus*, were closely related, while the tetraploid *R. glaucus* aligned with some putative blackberry × raspberry hybrids [42], suggesting this series defined by Focke is not monophyletic.

Clade XVI––Clade XVI contained *Rubus amabilis*, *Rubus maershanensis*, *Rubus* cf. *ptilocarpus*, etc., in our sampling. It was sister to the lineage containing Clade XVII–Clade XIX with strong support (ML BS = 100%, MP JK = 100%; Figure 1). Species included in Clade XVI are mainly distributed in the Himalayan region. Morphologically they share the following features: shrubs or subshrubs (*R. maershanensis*), flowers solitary or inflorescences with few flowers (2–6-flowered) and aggregate fruit usually red, glabrous or pubescent, not tomentose. Two well-supported subclades were identified. The first subclade was represented by *Rubus subornatus* var. *subornatus*, *R. subornatus* var. *melanadenus*, *Rubus sikkimensis*, *Rubus stans* and *R.* cf. *stans*, all usually with three-foliolate leaves. The second subclade contained *R. amabilis*, *R. maershanensis* and *R.* cf. *ptilocarpus* and was characterized by having 7–11-foliolate leaves.

Clade XVII––Clade XVII, composed of *Rubus pungens* var. *pungens*, *R. pungens* var. *ternatus* and *R. pungens* var. *oldhamii*, was resolved as sister to the monophyletic lineage formed by Clade XVIII and Clade XIX with maximum support (Figure 1). Generally, species of Clade XVII are widely distributed from Kashmir to Japan and exhibit complex morphological variability: prickles dense to sparse, glandular hairs present or absent and size of leaflets unstable. However, they differ in appearance from the other species of *Rubus* we sampled: shrubs, stems longer, climbing or trailing, with dense or sparse needle-like prickles (*R. pungens* var. *oldhamii* sometimes nearly unarmed), vegetative reproduction mainly by rooting at apex of stem, three–nine-foliolate leaves, abaxial surface of calyx with needle-like prickles, inflorescences terminal or axillary, 1-flowered or corymbose 2- to 4-flowered.

Clade XVIII––Clade XVIII included *R. biflorus* var. *biflorus*, *R.* cf. *biflorus* var. *adenophorus*, *R. biflorus* var. *pubescens*, *R. pseudopileatus* var. *glabratus*, *R.* cf. *pseudopileatus* var. *glabratus* and *R. trijugus* and was sister to Clade XIX with maximum support (Figure 1). Species contained in Clade XVIII occur in Asia and have the following features: shrubs, branchlets usually pruinose, pedicels distally inflated, leaves thick papyraceous or semi-leathery, aggregate fruit yellow or reddish yellow and densely gray tomentose (tomentum deciduous in R. biflorus and its varieties), flowers 1.5–3 cm in diameter and one to several in corymbose inflorescences.

Clade XIX––Clade XIX was sister to Clade XVIII and contained 20 species and seven varieties (Figure 1). All taxa of Clade XIX are shrubs occurring mainly in Asia. They have pink or purplish red petals (*Rubus mesogaeus* and *Rubus eucalyptus* have white or pink petals), which might be interpreted as morphological synapomorphies for this clade. Clade XIX can be divided into four subclades (Figure 1). The first contains *Rubus* cf. *cockburnianus*, *R. cockburnianus*, *R. eucalyptus* and *Rubus flosculosus* var. *etomentosus*, etc. with medium support (ML BS = 89%, MP JK = 73%). Most species of this subclade, except *R. eucalyptus* and *Rubus subinopertus*, have nearly purplish black aggregate fruit. *R.* cf. *cockburnianus*, *R. cockburnianus*, *R. flosculosus* var. *etomentosus*, together with *Rubus inopertus* var. *inopertus*, *R. inopertus* var. *echinocalyx*, *R. subinopertus*, all with 5–11-foliolate leaves, formed a lineage which was sister to the lineage composed of *R. eucalyptus*, *R. mesogaeus* and *Rubus subtibetanus*, which have 3- or 5-foliolate leaves. The second subclade contained *R. lasiostylus* var. *lasiostylus*, *R. lasiostylus* var. *villosus* and *Rubus wushanensis* with maximum support. Species of this subclade have broader stipules and bracts that are ovate-lanceolate, ovate or suborbicular. The third subclade contained *R. coreanus* var. *coreanus*, *R. coreanus* var. *tomentosus*, *Rubus niveus* and *Rubus thibetanus* with maximum support. The species of this subclade have 5–11 (or 13)-foliolate leaves and corymbose inflorescences. The fourth subclade contained *Rubus adenophorus*, *R. flosculosus* and *Rubus foliolosus*, etc. Members of this subclade exhibit significant morphological variability: three-foliolate leaves or five- or seven-foliolate leaves, inflorescences nearly corymbose, racemose or paniculate and plants with or without glandular hairs.

According to Focke’s [10,11] classification, most of the species in our study belong to *R*. subg. *Idaeobatus*. Our study showed that members of *R*. subg. *Idaeobatus sensu* Focke [10,11] are distributed among 13 clades (Clades IV–X, XIII and XV–XIX, Figure 1). The results showing that subgenus *Idaeobatus* is not monophyletic are consistent with the findings of previous studies [1,21,42,46,47,50,52,53]. Species of *R.* subg. *Idaeobatus* sect. *Pungentes sensu* Focke [10,11] occurring in Clade IX (*R. macilentus*), Clade XVI (*R. amabilis*, *R. maershanensis*, *R. sikkimensis*, *R. stans*), Clade XVII (*R. pungens*), Clade XVIII (*R. biflorus*) and Clade XIX (*R. eucalyptus*, *R. lasiostylus*) indicated that this section is not monophyletic. Similarly, *R.* subg. *Idaeobatus* sect. *Idaeanthi sensu* Focke [10,11] is not monophyletic as species of this section occurred in Clade IV (*R. ellipticus* var. *obcordatus*, *R. wallichianus*), Clade XVI (*R. subornatus*) and Clade XIX (*R. adenophorus*, *R. cockburnianus*, *R. flosculosus*, *Rubus idaeopsis*, *Rubus innominatus*, *R. inopertus*, *R. niveus*, *Rubus parvifolius*, *Rubus phoenicolasius*, *R. thibetanus*). *R.* sect. *Idaeobatus* subect. *Idaeanthi sensu* Lu and Yu [6] (*R. eucalyptus* and *R. niveus*) is paraphyletic, and *R.* sect. *Idaeobatus* subect. *Pungentes sensu* Lu and Yu [6] is not monophyletic as members of the latter subsection occurred in Clades IV (*R. wallichianus*), IX (*R. simplex*, *R. xanthocarpus*), XVI (*R. amabilis*, *R. maershanensis, R. sikkimensis, R. stans*), XVII (*R. pungens*) and XIX (*R. inopertus*). *R.* sect. *Idaeobatus* subsect. *Pileati sensu* Lu and Yu [6] is not monophyletic as members of this subsection occurred in Clades XVIII (*R. pseudopileatus* var. *glabratus*) and XIX (*R. subinopertus*). *R.* sect. *Idaeobatus* subect. *Stimulantes sensu* Lu and Yu [6] is not monophyletic as species of this subsection occurred in Clades IV (*R. ellipticus* var. *obcordatus*), XVI (*R. subornatus*), XVIII (*R. biflorus*) and XIX (*R. mesogaeus*, *R. parvifolius*, *R. phoenicolasius*, *R. thibetanus*). *R.* sect. *Idaeobatus* subect. *Thyrsidaei sensu* Lu and Yu [6] is paraphyletic as species of this subsection were embedded in Clade XIX (*R. adenophorus*, *R. cockburnianus*, *R. flosculosus*, *R. idaeopsis*, *R. innominatus*). These non-monophyletic sections and subsections indicate that morphological characteristics, such as whether inflorescences are paniculate or not, the indument of the abaxial surface of leaves and aggregate fruits, are not suitable for the classification of *R.* subg. *Idaeobatus*.

### 4.3. A Preliminary Classification Scheme of the Diploid Species of Rubus

**Figure 3:**
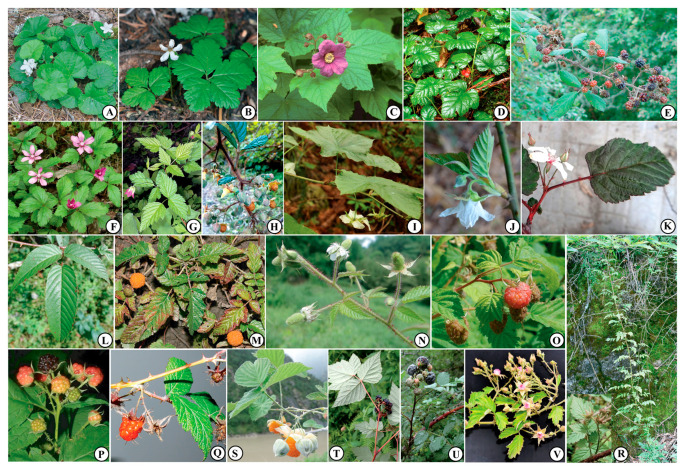
Representative species of *Rubus* from the 20 major lineages of the preliminary classification scheme proposed in the current study. (**A**) *R. repens* (Group 1, cited from Native Plant Trust, photo credit: Donald Cameron); (**B**) *R. pedatus* (Group 2, cited from Oregon Flora Image Project, photo credit: Gerald D. Carr); (**C**) *R. odoratus* (Group 3, photo credit: Sally Wasowski and Andy Wasowski, Lady Bird Johnson Wildflower Center); (**D**) *R. nivalis* (Group 4, cited from GBIF, photo credit: Tyson Ehlers); (**E**) *R. ulmifolius* (Group 5, cited from Flora-On, photo credit: Carlos Aguiar); (**F**) *R. arcticus* (Group 6, cited from Saxifaga Foundation–Images of European biodiversity, photo credit: Dirk Hilbers); (**G**) *R. spectabilis* (Group 7, cited from Oregon Flora Image Project, photo credit: Gerald D. Carr); (**H**) *R. ellipticus* var. *obcordatus* (Group 8); (**I**) *R. peltatus* (Group 9); (**J**) *R. corchorifolius* (Group 10); (**K**) *R. conduplicatus* (Group 11); (**L**) *R. columellaris* (Group 12); (**M**) *R. xanthocarpus* (Group 13); (**N**) *R. sumatranus* (Group 14); (**O**) *R. idaeus* (Group 15, cited from Saxifraga Foundation–Images of European biodiversity, photo credit: Jan Van der Straaten); (**P**) *R. occidentalis* (Group 16, cited from Discover Life, photo credit: Steven J. Baskauf); (**Q**) *R. subornatus* (Group 17); (**R**) *R. pungens* (Group 18); (**S**) *R. biflorus* (Group 19); (**T**) *R. mesogaeus* (Group 20); (**U**) *R. niveus* (Group 20); (**V**) *R. parvifolius* (Group 20).

According to our results, combined with previous phylogenetic analyses [1,21,37,42,46,47,49,50,52,53], a preliminary classification scheme of the diploid species of *Rubus* (Figure 3) is proposed herein. *R. paniculatus*, representing *R*. subg. *Malachobatus*, was excluded since its ploidy level is uncertain and it was not verified in this study. Before making formal taxonomic and nomenclatural decisions on an infrageneric classification, more samples and molecular data are needed to unravel and confirm the relationships and evolutionary history of these clades.

A preliminary classification scheme of the diploid species of *Rubus*.

Group 1

*Rubus repens* (L.) Kuntze (North America)

Group 2

*R. lasiococcus* A. Gray (North America)

*R. pedatus* Sm. (NE Asia, North America)

Group 3

*Rubus deliciosus* Torr. (North America)

*R. odoratus* L. (North America)

*R. parviflorus* Nutt. (North America)

*Rubus trilobus* Ser. (Mesoamerica)

Group 4

*Rubus nivalis* Douglas (North America)

Group 5

*R. allegheniensis* Porter (North America)

*R. argutus* Link (North America)

*R. canadensis* L. (North America)

*Rubus canescens* DC. (SW Asia, S and C Europe)

*Rubus coriifolius* Liebm. (Mesoamerican)

*Rubus cuneifolius* Pursh (North America)

*Rubus hispidus* L. (North America)

*Rubus robustus* C. Presl (South America)

*Rubus sanctus* Schreb. (N Africa, W Asia, SE Europe)

*Rubus setosus* Bigelow (North America)

*Rubus trivialis* Michx. (North America)

*Rubus ulmifolius* Schott (N Africa, Europe)

*Rubus urticifolius* Poir. (Mexico to S Tropical America)

Group 6

*R. arcticus* L. (Eurasia, North America)

*R. arcticus* L. subsp. *stellatus* (Sm.) B. Boivin (North America, Russian Far East)

*R. pubescens* Raf. (North America)

Group 7

*Rubus hawaiensis* A. Gray (Hawaii)

*Rubus spectabilis* Pursh (North America)

Group 8

*R. ellipticus* Sm. (Asia)

*R. ellipticus* Sm. var. *obcordatus* (Franch.) Focke (Asia)

*R. wallichianus* Wight et Arn. (Asia)

Group 9

*R. peltatus* Maxim. (China, Japan)

Group 10

*R. chingii* Hu (China, Japan)

*R. corchorifolius* L. f. (Asia)

*R. glabricarpus* Cheng (China)

*R. glabricarpus* Cheng var. *glabratus* C. Z. Zheng et Y. Y. Fang (China)

*R. palmatus* Thunb. (Japan, Korea)

Group 11

*R. conduplicatus* Duthie ex J. H. Veitch (China, Japan)

*R. crataegifolius* Bunge (E Asia)

*Rubus trifidus* Thunb. (Japan, Korea)

Group 12

*R. columellaris* Tutcher (China, Vietnam)

*R. impressinervus* F. P. Metcalf (China)

Group 13

*R. delavayi* Franch. (China)

*R. macilentus* Jacquem. ex Cambess. (Himalaya)

*R. simplex* Focke (China)

*R. xanthocarpus* Bureau et Franch. (China)

Group 14

*Rubus croceacanthus* H. Lév. (E and SE Asia)

*R. eustephanos* Focke (China)

*R. hirsutus* Thunb. (E Asia)

*Rubus illecebrosus* Focke (Japan)

*R. rosifolius* Sm. (Asia)

*R. sumatranus* Miq. (Asia)

*R. tsangii* Merr. (China)

Group 15

*Rubus idaeus* L. (Africa, Eurasia, North America)

*Rubus strigosus* Michx. (North America)

Group 16

*R. eriocarpus* Liebm. (Mesoamerica)

*R. leucodermis* Douglas ex Torr. et A. Gray (North America)

*R. occidentalis* L. (North America)

Group 17

*R. amabilis* Focke (China)

*R. maershanensis* Huan C. Wang et H. Sun (China)

*R. sikkimensis* Hook. f. (E Himalaya)

*R. stans* Focke (China)

*R. subornatus* Focke (China, Myanmar)

*R. subornatus* Focke var. *melanadenus* Focke (China)

Group 18

*R. pungens* Cambess. (Asia)

*R. pungens* Cambess. var. *oldhamii* (Miq.) Maxim. (E Asia)

*R. pungens* Cambess. var. *ternatus* Cardot (China)

Group 19

*R. biflorus* Buch.-Ham. ex Sm. (Asia)

*R. biflorus* Buch.-Ham. ex Sm. var. *pubescens* T. T. Yu et L. T. Lu (China)

*R. pseudopileatus* Cardot var. *glabratus* T. T. Yu et L. T. Lu (China)

*R. trijugus* Focke (China)

Group 20

*R. adenophorus* Rolfe (China)

*R. cockburnianus* Hemsl. (China)

*R. coreanus* Miq. (E Asia)

*R. coreanus* Miq. var. *tomentosus* Cardot (China)

*R. eucalyptus* Focke (China)

*R. flosculosus* Focke (China)

*R. flosculosus* Focke var. *etomentosus* T. T. Yu et L. T. Lu (China)

*R. foliolosus* D. Don (Asia)

*Rubus grandipaniculatus* T. T. Yu et L. T. Lu (China)

*R. idaeopsis* Focke (China)

*R. innominatus* S. Moore (China)

*R. innominatus* S. Moore var. *aralioides* (Hance) T. T. Yu et L. T. Lu (China)

*R. innominatus* S. Moore var. *kuntzeanus* (Hemsl.) L. H. Bailey (China)

*R. inopertus* (Focke) Focke (China, Vietnam)

*R. inopertus* (Focke) Focke var. *echinocalyx* Cardot (China)

*R. lasiostylus* Focke (China)

*R. lasiostylus* Focke var. *villosus* Cardot (China)

*R. mesogaeus* Focke. (Asia)

*R. niveus* Thunb. (Asia)

*R. parvifolius* L. (E Asia, Australia)

*R. parvifolius* L. var. *adenochlamys* (Focke) Migo (China, Japan)

*R. phoenicolasius* Maxim. (E Asia)

*Rubus subcoreanus* T. T. Yu et L. T. Lu (China)

*R. subinopertus* T. T. Yu et L. T. Lu (China)

*R. subtibetanus* Hand.-Mazz. (China)

*R. thibetanus* Franch. (China)

*R. wushanensis* T. T. Yu et L. T. Lu (China)

Key to the Groups

1a. Flowers of two kinds, essentially petaliferous staminate flowers, usually with pistils abortive, fertile apetalous flowers and with few stamens; drupelets 5–10, nearly dry; low stoloniferous herbs with simple, unlobed, reniform leaves …………….…… Group 1

1b. Flowers of only one kind (in dioecious or polygamodioecious species only slightly dissimilar), mostly petaliferous, sometimes apetalous but bisexual; drupelets many or sometimes several, often fleshy; shrubs, subshrubs or herbs.

2a. Styles club-shaped; stigmas slightly 2-lobed; receptacle flat; unarmed shrubs with exfoliating bark and simple digitately ribbed and lobed leaves ……….………… Group 3

2b. Styles filiform; stigmas capitate; receptacle convex, hemispheric or nipple-shaped; stems unarmed, bristly or prickly; leaves simple or compound.

3a. Stem herbaceous, never prickly, rarely bristly; stipules broad, free or nearly so; floral branches arising directly from rootstock or from stolons.

4a. Filaments filiform, not dilated ……………………………………………… Group 2

4b. Filaments dilated, laminar ……………………………………………..…… Group 6

3b. Stem more or less woody, usually prickly; if unarmed or merely bristly, the stipules narrow and more or less adnate to the petiole.

5a. Stipules broad, nearly free, at junction of stem and petiole; plants with needle-like prickles or bristles; subshrubs, stems creeping ………………………………..…… Group 4

5b. Stipules narrow, linear-lanceolate or subulate, more or less adnate to the petiole; plants with or without needle-like prickles or bristles; shrubs, subshrubs or herblike.

6a. Drupelets remaining on fleshy receptacle at maturity or falling together with receptacle, or falling separately ………………………………………………..………..Group 5

6b. Drupelets united into a thimble-shaped aggregate fruit, falling off entire from dry receptacle.

7a. Flowers solitary or few, large, 2–3.5 cm in diameter; petals pink or magenta, much exceeding sepals; filaments laminar; leaves ternate, papyraceous ………..……… Group 7

7b. Flowers solitary or in inflorescences, small to large, if large (> 3 cm in diameter), the petals white; petals white, pink or purplish; leaves simple or compound, papyraceous or leathery.

8a. Plants with dense, spreading, reddish brown or purplish brown mostly eglandular bristles, 3–7 mm long; aggregate fruit golden yellow or reddish yellow; leaves three-foliolate ……………………………………………………………………………….… Group 8

8b. Plants usually without dense, spreading eglandular bristles; aggregate fruit color varied; leaves simple, 3–11 (or 13)-foliolate.

9a. Leaves leathery ……………………………………………………………… Group 12

9b. Leaves papyraceous or membranous.

10a. Leaves simple.

11a. Leaves peltate; carpels ca. 100 or slightly more; aggregate fruit terete or cylindrical …………………………………………………………………………….………..… Group 9

11b. Leaves not peltate; carpels ca. 10–60, rarely slightly more; aggregate fruit subglobose or ovoid-globose.

12a. Flowers solitary ………………………………………………………….… Group 10

12b. Flowers in nearly corymbiform inflorescences ……………………….… Group 11

10b. Leaves compound.

13a. Carpels ca. 100 or more, usually inserted on stipitate torus…………… Group 14

13b. Carpels 10–70 or slightly more, inserted on sessile torus.

14a. Subshrubs or nearly so; three-foliolate leaves; semi-leathery, terminal leaflet much or slightly longer than lateral leaflets; petals white; fruit orange (*R. macilentus* with orange or red fruit) …………………………………………………………………… Group 13

14b. Shrubs, if nearly subshrubs, leaflets more than three or petals pink or purplish; leaves 3–11-foliolate; petals white, pink, purplish; fruit red or black or sometimes orange.

15a. Stems scrambling or trailing, 1–3 m long, with dense or sparse needle-like prickles; abaxial surface of calyx with dense, straight needle-like prickles; leaves abaxially pubescent or nearly glabrous ……………………………………….…………….…… Group 18

15b. Stems mostly erect or arching, sometimes scandent or trailing, mostly without needle-like prickles; calyx unarmed or its prickles mostly not needle-like; leaves abaxially tomentose, pubescent or glabrous.

16a. Petals pink or purplish red (*R. mesogaeus* and *R. eucalyptus* with white and pink petals); mainly Asian, especially in China ………………………………………… Group 20

16b. Petals white (*R. subornatus*, *R. subornatus* var. *melanadenus* and *R. sikkimensis* with purplish red petals); occurring in Africa, Eurasia and North America.

17a. Aggregate fruit densely gray tomentose, yellow or reddish yellow; branchlets pruinose; pedicels distally inflated ………………………………………………… Group 19

17b. Aggregate fruit mostly glabrous or pilose, not gray tomentose, dark purplish, black, red, orangish red or sometimes yellow; branchlets pruinose or not.

18a. Aggregate fruit usually dark purplish or black; leaves palmately compound or ternate ………………………………………………………………………………… Group 16

18b. Aggregate fruit red, orangish red, or yellow; leaves pinnately compound or ternate.

19a. Native to Himalayan regions …………………………………………… Group 17

19b. Native to North America (*R. strigosus*), or Africa, Eurasia and North America (*R. idaeus*) …………………………………………………………………………….…… Group 15

## 5. Conclusions

The taxonomy of *Rubus* is challenging and the phylogenetic relationships within *Rubus* remain to be clarified. This study inferred the phylogeny of *Rubus* with emphasis on diploid species based on GBS data with comprehensive taxon sampling. Our results provided useful information for deducing the phylogeny of *Rubus*, especially providing important insights into the evolution of *Rubus* in China. We reconfirmed that *R.* subg. *Idaeobatus*, recognized by Focke, is not monophyletic. We found that characteristics such as leathery or papyraceous leaves may be of some use in classifying the raspberries. Based on our results, and combined with previous phylogenetic analyses, a preliminary classification scheme of the diploid species of *Rubus* is proposed here. Further studies, however, including more samples and additional molecular data, are still needed to better unravel and confirm the complicated evolutionary history of *Rubus*.

## Data Availability

All data from this study are available in the manuscript and Supplementary Materials.

## Supplementary Materials

The following supporting information can be downloaded at: https://www.mdpi.com/article/10.3390/genes14061152/s1, Table S1: Quality statistics of sequencing data of 190 samples; Table S2: Statistics of sequencing depth and coverage of nuclear genome of 190 samples; Table S3: Statistics of sequencing depth and coverage of chloroplast genome of 190 samples.

## Appendix A

**Table A1:** List of taxa sampled with information related to taxonomy, ploidy and vouchers.

Taxon	Subgeneric Classification of Focke [9, 10,11]	Sectional Classification of Yu and Lu [83], Lu and Yu [6], and Lu and Boufford [7]	Ploidy Level	Voucher
*Rubus odoratus* L.	*R.* subg. *Anoplobatus*	—	2*x*	USA: Massachusetts, Northampton, *Ano. s. n.* (BR, Living Plant Collections Accession 19941489-35)
*R. parviflorus* Nutt.	*R.* subg. *Anoplobatus*	—	2*x*	USA: *W. R. Smith 29200* (MO)
*R. arcticus* L. subsp. *stellatus* (Sm.) B. Boivin	*R.* subg. *Cylactis*	—	2*x*	USA: Alaska, Wasilla, *K. Hummer et C. I. Wright s. n.* (NPGS, Accession PI 606535)
*R. pubescens* Raf.	—	—	2*x*	USA: *D. Atha 7040* (MO)
*R. simplex* Focke	*R.* subg. *Cylactis*	*R.* sect. *Idaeobatus*	2*x*	China: Sichuan, Leibo, *X. H. Xiong 2036* (CDBI)
*R. xanthocarpus* Bureau et Franch. 1	*R.* subg. *Cylactis*	*R.* sect. *Idaeobatus*	2*x*	China: Sichuan, Baoxing, *X. H. Xiong 1159* (CDBI)
*R. xanthocarpus* Bureau et Franch. 2	*R.* subg. *Cylactis*	*R.* sect. *Idaeobatus*	2*x*	China: Sichuan, Kangding, *X. H. Xiong 1316* (CDBI)
*R. xanthocarpus* Bureau et Franch. 3	*R.* subg. *Cylactis*	*R.* sect. *Idaeobatus*	2*x*	China: Sichuan, Baoxing, *X. H. Xiong 2002* (CDBI)
*R. xanthocarpus* Bureau et Franch. 4	*R.* subg. *Cylactis*	*R.* sect. *Idaeobatus*	2*x*	China: Sichuan, Kangding, *X. H. Xiong 2020* (CDBI)
*R. lasiococcus* A. Gray	*R.* subg. *Dalibarda*	—	2*x*	USA: Oregon, Deschutes County, *O. L. Jahn s. n.* (NPGS, Accession PI 553659)
*R. pedatus* Sm.	*R.* subg. *Dalibarda*	—	2*x*	USA: Alaska, Sitka, *H. Chambers s. n.* (NPGS, Accession PI618503)
*R. adenophorus* Rolfe 1	*R.* subg. *Idaeobatus*	*R.* sect. *Idaeobatus*	2*x*	China: Zhejiang, Qingyuan, *X. H. Xiong 1967* (CDBI)
*R. adenophorus* Rolfe 2	*R.* subg. *Idaeobatus*	*R.* sect. *Idaeobatus*	2*x*	China: Zhejiang, Qingyuan, *X. H. Xiong 1968* (CDBI)
*R. amabilis* Focke 1	*R.* subg. *Idaeobatus*	*R.* sect. *Idaeobatus*	2*x*	China: Sichuan, Baoxing, *X. H. Xiong 1146-3* (CDBI)
*R. amabilis* Focke 2	*R.* subg. *Idaeobatus*	*R.* sect. *Idaeobatus*	2*x*	China: Sichuan, Baoxing, *X. H. Xiong 1146-7* (CDBI)
*R. amabilis* Focke 3	*R.* subg. *Idaeobatus*	*R.* sect. *Idaeobatus*	2*x*	China: Sichuan, Shimian, *X. H. Xiong 1863* (CDBI)
*R. amabilis* Focke 4	*R.* subg. *Idaeobatus*	*R.* sect. *Idaeobatus*	2*x*	China: Sichuan, Wenchuan, *X. H. Xiong 1870* (CDBI)
*R. biflorus* Buch.-Ham. ex Sm. 1	*R.* subg. *Idaeobatus*	*R.* sect. *Idaeobatus*	2*x*	China: Sichuan, Xiaojin, *X. H. Xiong 1860* (CDBI)
*R. biflorus* Buch.-Ham. ex Sm. 2	*R.* subg. *Idaeobatus*	*R.* sect. *Idaeobatus*	2*x*	China: Sichuan, Yajiang, *X. H. Xiong 2156* (CDBI)
*R. biflorus* Buch.-Ham. ex Sm. var. *pubescens* T. T. Yu et L. T. Lu	—	*R.* sect. *Idaeobatus*	NA	China: Sichuan, Muli, *X. H. Xiong 1365* (CDBI)
*R.* cf. *biflorus* Buch.-Ham. ex Sm. var. *adenophorus* Franch.	—	*R.* sect. *Idaeobatus*	NA	China: Xizang, Linzhi, *X. H. Xiong 1054* (CDBI)
*R. chingii* Hu 1	—	*R.* sect. *Idaeobatus*	2*x*	China: Zhejiang, Qingyuan, *X. H. Xiong 1966* (CDBI)
*R. chingii* Hu 2	—	*R.* sect. *Idaeobatus*	2*x*	China: Anhui, Huangshan, *X. H. Xiong 831* (CDBI)
*R. cockburnianus* Hemsl.	*R.* subg. *Idaeobatus*	*R.* sect. *Idaeobatus*	2*x*	China: Sichuan, Maoxian, *X. F. Gao* et al. *4983* (CDBI)
*R.* cf. *cockburnianus* Hemsl.	*R.* subg. *Idaeobatus*	*R.* sect. *Idaeobatus*	2*x*	China: Xizang, Bomi, *Y. D. Gao et M. Li YLZB0219* (CDBI)
*R. columellaris* Tutcher 1	—	*R.* sect. *Idaeobatus*	2*x*	China: Xizang, Motuo, *X. H. Xiong 1389* (CDBI)
*R. columellaris* Tutcher 2	—	*R.* sect. *Idaeobatus*	2*x*	China: Jiangxi, Ganzhou, *X. H. Xiong 1735* (CDBI)
*R. columellaris* Tutcher 3	—	*R.* sect. *Idaeobatus*	2*x*	China: Sichuan, *X. H. Xiong 1789* (CDBI)
*R. columellaris* Tutcher 4	—	*R.* sect. *Idaeobatus*	2*x*	China: Jiangxi, Jinggangshan, *X. H. Xiong 1970* (CDBI)
*R. conduplicatus* Duthie ex J. H. Veitch 1 (*R. trianthus* Focke)	*R.* subg. *Idaeobatus*	*R.* sect. *Idaeobatus*	2*x*	China: Jiangxi, Lushan, *X. H. Xiong 1648* (CDBI)
*R. conduplicatus* Duthie ex J. H. Veitch 2	*R.* subg. *Idaeobatus*	*R.* sect. *Idaeobatus*	2*x*	China: Zhejiang, Wenzhou, *X. H. Xiong 1836-3* (CDBI)
*R. conduplicatus* Duthie ex J. H. Veitch 3	*R.* subg. *Idaeobatus*	*R.* sect. *Idaeobatus*	2*x*	China: Zhejiang, Qingyuan, *X. H. Xiong 1912* (CDBI)
*R. conduplicatus* Duthie ex J. H. Veitch 4	*R.* subg. *Idaeobatus*	*R.* sect. *Idaeobatus*	2*x*	China: Zhejiang, Qingyuan, *X. H. Xiong 1964* (CDBI)
*R. corchorifolius* L. f. 1	*R.* subg. *Idaeobatus*	*R.* sect. *Idaeobatus*	2*x*	China: Jiangxi, Lushan, *X. H. Xiong 1634* (CDBI)
*R. corchorifolius* L. f. 2	*R.* subg. *Idaeobatus*	*R.* sect. *Idaeobatus*	2*x*	China: Jiangxi, Ganzhou, *X. H. Xiong 1733* (CDBI)
*R. corchorifolius* L. f. 3	*R.* subg. *Idaeobatus*	*R.* sect. *Idaeobatus*	2*x*	China: Yunnan, *X. H. Xiong 1742* (CDBI)
*R. corchorifolius* L. f. 4	*R.* subg. *Idaeobatus*	*R.* sect. *Idaeobatus*	2*x*	China: Zhejiang, Wenzhou, *X. H. Xiong 1828-2* (CDBI)
*R. corchorifolius* L. f. 5	*R.* subg. *Idaeobatus*	*R.* sect. *Idaeobatus*	2*x*	China: Zhejiang, Qingyuan, *X. H. Xiong 1918* (CDBI)
*R. coreanus* Miq.	*R.* subg. *Idaeobatus*	*R.* sect. *Idaeobatus*	2*x*	China: Shaanxi, *Xiong s. n.* (CDBI)
*R. coreanus* Miq. var. *tomentosus* Cardot 1	—	*R.* sect. *Idaeobatus*	NA	China: Chongqing, Wushan, *X. H. Xiong 1173* (CDBI)
*R. coreanus* Miq. var. *tomentosus* Cardot 2	—	*R.* sect. *Idaeobatus*	NA	China: Chongqing, Wushan, *X. H. Xiong 1176* (CDBI)
*R. coreanus* Miq. var. *tomentosus* Cardot 3	—	*R.* sect. *Idaeobatus*	NA	China: Sichuan, *X. H. Xiong 1771* (CDBI)
*R. crataegifolius* Bunge	*R.* subg. *Idaeobatus*	*R.* sect. *Idaeobatus*	2*x*, 3*x*, 4*x*	China: Beijing, *X. H. Xiong 1991* (CDBI)
*R. delavayi* Franch.	*R.* subg. *Idaeobatus*	*R.* sect. *Idaeobatus*	NA	China: Yunnan, Shizong, *X. H. Xiong 1824* (CDBI)
*R. ellipticus* Sm. var. *obcordatus* (Franch.) Focke 1	*R.* subg. *Idaeobatus*	*R.* sect. *Idaeobatus*	2*x*	China: Xizang, Motuo, *X. H. Xiong 1427* (CDBI)
*R. ellipticus* Sm. var. *obcordatus* (Franch.) Focke 2	*R.* subg. *Idaeobatus*	*R.* sect. *Idaeobatus*	2*x*	China: Yunnan, Maguan, *X. H. Xiong 1709* (CDBI)
*R. ellipticus* Sm. var. *obcordatus* (Franch.) Focke 3	*R.* subg. *Idaeobatus*	*R.* sect. *Idaeobatus*	2*x*	China: Yunnan, *X. H. Xiong 1737* (CDBI)
*R. ellipticus* Sm. var. *obcordatus* (Franch.) Focke 4	*R.* subg. *Idaeobatus*	*R.* sect. *Idaeobatus*	2*x*	China: Sichuan, *X. H. Xiong 1758* (CDBI)
*R. eucalyptus* Focke 1	*R.* subg. *Idaeobatus*	*R.* sect. *Idaeobatus*	NA	China: Sichuan, Kangding, *X. H. Xiong 1304* (CDBI)
*R. eucalyptus* Focke 2	*R.* subg. *Idaeobatus*	*R.* sect. *Idaeobatus*	NA	China: Sichuan, *X. H. Xiong 1839* (CDBI)
*R. eucalyptus* Focke 3	*R.* subg. *Idaeobatus*	*R.* sect. *Idaeobatus*	NA	China: Sichuan, Dayi, *X. H. Xiong 1856* (CDBI)
*R. eucalyptus* Focke 4	*R.* subg. *Idaeobatus*	*R.* sect. *Idaeobatus*	NA	China: Sichuan, *X. H. Xiong 1993* (CDBI)
*R. eustephanos* Focke 1	*R.* subg. *Idaeobatus*	*R.* sect. *Idaeobatus*	2*x*	China: Sichuan, Chengdu, *X. H. Xiong 1534* (CDBI)
*R. eustephanos* Focke 2	*R.* subg. *Idaeobatus*	*R.* sect. *Idaeobatus*	2*x*	China: Sichuan, *X. H. Xiong 1784* (CDBI)
*R. flosculosus* Focke 1	*R.* subg. *Idaeobatus*	*R.* sect. *Idaeobatus*	2*x*	China: Zhejiang, Xianju, *S. Q. Xu 1299* (CDBI)
*R. flosculosus* Focke 2	*R.* subg. *Idaeobatus*	*R.* sect. *Idaeobatus*	2*x*	China: Zhejiang, Suichang, *S. Q. Xu 1344* (CDBI)
*R. flosculosus* Focke var. *etomentosus* T. T. Yu et L. T. Lu	—	*R.* sect. *Idaeobatus*	NA	China: Sichuan, Baoxing, *X. H. Xiong 2014* (CDBI)
*R. foliolosus* D. Don 1	*R.* subg. *Idaeobatus*	*R.* sect. *Idaeobatus*	2*x*, 4*x*	China: Yunnan, Malipo, *X. H. Xiong 1712* (CDBI)
*R. foliolosus* D. Don 2	*R.* subg. *Idaeobatus*	*R.* sect. *Idaeobatus*	2*x*, 4*x*	China: Yunnan, *X. H. Xiong 1744* (CDBI)
*R. glabricarpus* Cheng 1	—	*R.* sect. *Idaeobatus*	NA	China: Zhejiang, Wenzhou, *X. H. Xiong 1838-1* (CDBI)
*R. glabricarpus* Cheng 2	—	*R.* sect. *Idaeobatus*	NA	China: Zhejiang, Wenzhou, *X. H. Xiong 1838-3* (CDBI)
*R. glabricarpus* Cheng 3	—	*R.* sect. *Idaeobatus*	NA	China: Zhejiang, Qingyuan, *X. H. Xiong 1947* (CDBI)
*R. glabricarpus* Cheng 4	—	*R.* sect. *Idaeobatus*	NA	China: Zhejiang, Qingyuan, *X. H. Xiong 1949* (CDBI)
*R. glabricarpus* Cheng var. *glabratus* C. Z. Zheng et Y. Y. Fang	—	*R.* sect. *Idaeobatus*	NA	China: Zhejiang, Qingyuan, *X. H. Xiong 1933* (CDBI)
*R.* cf. *glabricarpus* Cheng var. *glabratus* C. Z. Zheng et Y. Y. Fang	—	*R.* sect. *Idaeobatus*	NA	China: Fujian, Wuyishan, *M. Li LMWYS-45* (CDBI)
*R. grandipaniculatus* T. T. Yu et L. T. Lu 1	—	*R.* sect. *Idaeobatus*	NA	China: Chongqing, Wuxi, *X. H. Xiong 2080* (CDBI)
*R. grandipaniculatus* T. T. Yu et L. T. Lu 2	—	*R.* sect. *Idaeobatus*	NA	China: Chongqing, Wuxi, *X. H. Xiong 2087* (CDBI)
*R. hirsutus* Thunb. 1	—	*R.* sect. *Idaeobatus*	2*x*	China: Zhejiang, Hangzhou, *X. H. Xiong 1594* (CDBI)
*R. hirsutus* Thunb. 2	—	*R.* sect. *Idaeobatus*	2*x*	China: Jiangxi, Lushan, *X. H. Xiong 1635* (CDBI)
*R. hirsutus* Thunb. 3	—	*R.* sect. *Idaeobatus*	2*x*	China: Jiangxi, Ganzhou, *X. H. Xiong 1734* (CDBI)
*R. hirsutus* Thunb. 4	—	*R.* sect. *Idaeobatus*	2*x*	China: Zhejiang, Wenzhou, *X. H. Xiong 1837-1* (CDBI)
*R. hirsutus* Thunb. 5	—	*R.* sect. *Idaeobatus*	2*x*	China: Jiangxi, Jinggangshan, *X. H. Xiong 1983* (CDBI)
*R. idaeopsis* Focke 1	*R.* subg. *Idaeobatus*	*R.* sect. *Idaeobatus*	2*x*	China: Sichuan, Luzhou, *X. H. Xiong 1627* (CDBI)
*R. idaeopsis* Focke 2	*R.* subg. *Idaeobatus*	*R.* sect. *Idaeobatus*	2*x*	China: Sichuan, Luzhou, *X. H. Xiong 1628* (CDBI)
*R. impressinervus* F. P. Metcalf 1	—	*R.* sect. *Idaeobatus*	NA	China: Zhejiang, Qingyuan, *X. H. Xiong 1930* (CDBI)
*R. impressinervus* F. P. Metcalf 2	—	*R.* sect. *Idaeobatus*	NA	China: Zhejiang, Qingyuan, *X. H. Xiong 1935* (CDBI)
*R. innominatus* S. Moore 1	*R.* subg. *Idaeobatus*	*R.* sect. *Idaeobatus*	2*x*	China: Chongqing, Wuxi, *Ano. THP-WX-1601* (CDBI)
*R. innominatus* S. Moore 2	*R.* subg. *Idaeobatus*	*R.* sect. *Idaeobatus*	2*x*	China: Chongqing, Chengkou, *X. H. Xiong 1220* (CDBI)
*R. innominatus* S. Moore 3	*R.* subg. *Idaeobatus*	*R.* sect. *Idaeobatus*	2*x*	China: Sichuan, Kangding, *X. H. Xiong 1300* (CDBI)
*R. innominatus* S. Moore 4	*R.* subg. *Idaeobatus*	*R.* sect. *Idaeobatus*	2*x*	China: Sichuan, *X. H. Xiong 1788* (CDBI)
*R. innominatus* S. Moore var. *aralioides* (Hance) T. T. Yu et L. T. Lu	*R.* subg. *Idaeobatus*	*R.* sect. *Idaeobatus*	NA	China: Jiangxi, Lushan, *X. H. Xiong 1645* (CDBI)
*R. innominatus* S. Moore var. *kuntzeanus* (Hemsl.) L. H. Bailey	*R.* subg. *Idaeobatus*	*R.* sect. *Idaeobatus*	2*x*	China: Sichuan, Xuyong, *X. H. Xiong 1551* (CDBI)
*R. inopertus* (Focke) Focke	*R.* subg. *Idaeobatus*	*R.* sect. *Idaeobatus*	2*x*	China: Jiangxi, Jinggangshan, *X. H. Xiong 1985* (CDBI)
*R. inopertus* (Focke) Focke var. *echinocalyx* Cardot	—	*R.* sect. *Idaeobatus*	NA	China: Sichuan, Xuyong, *X. H. Xiong 1562* (CDBI)
*R. lasiostylus* Focke	*R.* subg. *Idaeobatus*	*R.* sect. *Idaeobatus*	2*x*	China: Shaanxi, Xian, *C. Zhang et S. Z. Yang zc12087* (CDBI)
*R. lasiostylus* Focke var. *villosus* Cardot	—	*R.* sect. *Idaeobatus*	NA	China: Chongqing, Chengkou, *X. H. Xiong 1209* (CDBI)
*R. leucodermis* Douglas ex Hook.	*R.* subg. *Idaeobatus*	—	2*x*	Canada: British Columbia, *H. A. Daubeny s. n.* (NPGS, Accession PI 618562)
*R. macilentus* Jacquem. ex Cambess. 1	*R.* subg. *Idaeobatus*	*R.* sect. *Idaeobatus*	2*x*	China: Xizang, Motuo, *X. H. Xiong 1418* (CDBI)
*R. macilentus* Jacquem. ex Cambess. 2	*R.* subg. *Idaeobatus*	*R.* sect. *Idaeobatus*	2*x*	China: Sichuan, Shimian, *X. H. Xiong 1430* (CDBI)
*R. macilentus* Jacquem. ex Cambess. 3	*R.* subg. *Idaeobatus*	*R.* sect. *Idaeobatus*	2*x*	China: Sichuan, Chengdu, *X. H. Xiong 1535* (CDBI)
*R. macilentus* Jacquem. ex Cambess. 4	*R.* subg. *Idaeobatus*	*R.* sect. *Idaeobatus*	2*x*	China: Sichuan, *X. H. Xiong 1763* (CDBI)
*R. maershanensis* Huan C. Wang et H. Sun 1 (*R. lutescens* Franch.)	*R.* subg. *Idaeobatus*	*R.* sect. *Idaeobatus*	NA	China: Sichuan, Kangding, *X. H. Xiong 1259* (CDBI)
*R. maershanensis* Huan C. Wang et H. Sun 2	*R.* subg. *Idaeobatus*	*R.* sect. *Idaeobatus*	NA	China: Sichuan, Muli, *X. H. Xiong 1362* (CDBI)
*R. maershanensis* Huan C. Wang et H. Sun 3	*R.* subg. *Idaeobatus*	*R.* sect. *Idaeobatus*	NA	China: Sichuan, Kangding, *X. H. Xiong 1786* (CDBI)
*R. mesogaeus* Focke 1	*R.* subg. *Idaeobatus*	*R.* sect. *Idaeobatus*	2*x*	China: Sichuan, Baoxing, *X. H. Xiong 1080* (CDBI)
*R. mesogaeus* Focke 2	*R.* subg. *Idaeobatus*	*R.* sect. *Idaeobatus*	2*x*	China: Xizang, Cuona, *X. H. Xiong 1575* (CDBI)
*R. mesogaeus* Focke 3	*R.* subg. *Idaeobatus*	*R.* sect. *Idaeobatus*	2*x*	China: Sichuan, Shimian, *X. H. Xiong 1861* (CDBI)
*R. mesogaeus* Focke 4	*R.* subg. *Idaeobatus*	*R.* sect. *Idaeobatus*	2*x*	China: Sichuan, Baoxing, *X. H. Xiong 2007* (CDBI)
*R. mesogaeus* Focke 5	*R.* subg. *Idaeobatus*	*R.* sect. *Idaeobatus*	2*x*	China: Sichuan, Jinyang, *X. H. Xiong 2030* (CDBI)
*R. niveus* Thunb. 1	*R.* subg. *Idaeobatus*	*R.* sect. *Idaeobatus*	2*x*	China: Sichuan, Kangding, *X. H. Xiong 1268* (CDBI)
*R. niveus* Thunb. 2	*R.* subg. *Idaeobatus*	*R.* sect. *Idaeobatus*	2*x*	China: Sichuan, Yajiang, *X. H. Xiong 1345* (CDBI)
*R. niveus* Thunb. 3	*R.* subg. *Idaeobatus*	*R.* sect. *Idaeobatus*	2*x*	China: Yunnan, Malipo, *X. H. Xiong 1718* (CDBI)
*R. niveus* Thunb. 4	*R.* subg. *Idaeobatus*	*R.* sect. *Idaeobatus*	2*x*	China: Yunnan, *X. H. Xiong 1741* (CDBI)
*R. niveus* Thunb. 5	*R.* subg. *Idaeobatus*	*R.* sect. *Idaeobatus*	2*x*	China: Sichuan, *X. H. Xiong 1787* (CDBI)
*R. niveus* Thunb. 6	*R.* subg. *Idaeobatus*	*R.* sect. *Idaeobatus*	2*x*	China: Sichuan, Luding, *X. H. Xiong 1862* (CDBI)
*R. occidentalis* L.	*R.* subg. *Idaeobatus*	—	2*x*	USA: Oregon, *Ano. s. n.* (NPGS, Accession PI 672663)
*R. parvifolius* L. 1	*R.* subg. *Idaeobatus*	*R.* sect. *Idaeobatus*	2*x*, 3*x*, 4*x*	China: Sichuan, Chengdu, *X. H. Xiong 1528* (CDBI)
*R. parvifolius* L. 2	*R.* subg. *Idaeobatus*	*R.* sect. *Idaeobatus*	2*x*, 3*x*, 4*x*	China: Zhejiang, Hangzhou, *X. H. Xiong 1591* (CDBI)
*R. parvifolius* L. 3	*R.* subg. *Idaeobatus*	*R.* sect. *Idaeobatus*	2*x*, 3*x*, 4*x*	China: Anhui, Susong, *X. H. Xiong 1703* (CDBI)
*R. parvifolius* L. 4	*R.* subg. *Idaeobatus*	*R.* sect. *Idaeobatus*	2*x*, 3*x*, 4*x*	China: Sichuan, Luzhou, *X. H. Xiong 1804* (CDBI)
*R. parvifolius* L. var. *adenochlamys* (Focke) Migo	*R.* subg. *Idaeobatus*	*R.* sect. *Idaeobatus*	NA	China: Shaanxi, Taibai, *X. H. Xiong 1333* (CDBI)
*R. peltatus* Maxim. 1	*R.* subg. *Idaeobatus*	*R.* sect. *Idaeobatus*	2*x*	China: Zhejiang, Qingyuan, *X. H. Xiong 1938* (CDBI)
*R. peltatus* Maxim. 2	*R.* subg. *Idaeobatus*	*R.* sect. *Idaeobatus*	2*x*	China: Zhejiang, Qingyuan, *X. H. Xiong 1942* (CDBI)
*R. peltatus* Maxim. 3	*R.* subg. *Idaeobatus*	*R.* sect. *Idaeobatus*	2*x*	China: Jiangxi, Jinggangshan, *X. H. Xiong 1987* (CDBI)
*R. pentagonus* Wall.	*R.* subg. *Idaeobatus*	*R.* sect. *Idaeobatus*	4*x*	China: Xizang, Motuo, *X. H. Xiong 1409* (CDBI)
*R. pentagonus* Wall. var. *eglandulosus* T. T. Yu et L. T. Lu 1	—	*R.* sect. *Idaeobatus*	NA	China: Xizang, Motuo, *B. Xu et X. H. Xiong YLZB1151* (CDBI)
*R. pentagonus* Wall. var. *eglandulosus* T. T. Yu et L. T. Lu 2	—	*R.* sect. *Idaeobatus*	NA	China: Xizang, Motuo, *B. Xu et X. H. Xiong YLZB1174* (CDBI)
*R. pentagonus* Wall. var. *eglandulosus* T. T. Yu et L. T. Lu 3	—	*R.* sect. *Idaeobatus*	NA	China: Xizang, Motuo, *X. H. Xiong 1406* (CDBI)
*R. phoenicolasius* Maxim. 1	*R.* subg. *Idaeobatus*	*R.* sect. *Idaeobatus*	2*x*	China: Shaanxi, Hanzhong, *X. H. Xiong 1322* (CDBI)
*R. phoenicolasius* Maxim. 2	*R.* subg. *Idaeobatus*	*R.* sect. *Idaeobatus*	2*x*	China: Shaanxi, Taibai, *X. H. Xiong 1334* (CDBI)
*R. pseudopileatus* Cardot var. *glabratus* T. T. Yu et L. T. Lu 1	—	*R.* sect. *Idaeobatus*	NA	China: Sichuan, Baoxing, *X. H. Xiong 1147-1* (CDBI)
*R. pseudopileatus* Cardot var. *glabratus* T. T. Yu et L. T. Lu 2	—	*R.* sect. *Idaeobatus*	NA	China: Sichuan, Baoxing, *X. H. Xiong 1147-27* (CDBI)
*R. pseudopileatus* Cardot var. *glabratus* T. T. Yu et L. T. Lu 3	—	*R.* sect. *Idaeobatus*	NA	China: Sichuan, Baoxing, *X. H. Xiong 1147-8* (CDBI)
*R.* cf. *pseudopileatus* Cardot var. *glabratus* T. T. Yu et L. T. Lu	—	*R.* sect. *Idaeobatus*	NA	China: Sichuan, Luding, *X. H. Xiong 1868* (CDBI)
*R.* cf. *ptilocarpus* T. T. Yu et L. T. Lu 1	—	*R.* sect. *Idaeobatus*	NA	China: Sichuan, Baoxing, *X. H. Xiong 2003* (CDBI)
*R.* cf. *ptilocarpus* T. T. Yu et L. T. Lu 2	—	*R.* sect. *Idaeobatus*	NA	China: Sichuan, Baoxing, *X. H. Xiong 2005* (CDBI)
*R. pungens* Cambess. 1	*R.* subg. *Idaeobatus*	*R.* sect. *Idaeobatus*	2*x*	China: Sichuan, Baoxing, *X. H. Xiong 1162* (CDBI)
*R. pungens* Cambess. 2	*R.* subg. *Idaeobatus*	*R.* sect. *Idaeobatus*	2*x*	China: Chongqing, Wushan, *X. H. Xiong 1186* (CDBI)
*R. pungens* Cambess. 3	*R.* subg. *Idaeobatus*	*R.* sect. *Idaeobatus*	2*x*	China: Chongqing, Chengkou, *X. H. Xiong 1200* (CDBI)
*R. pungens* Cambess. 4	*R.* subg. *Idaeobatus*	*R.* sect. *Idaeobatus*	2*x*	China: Xizang, Milin, *B. Xu et X. M. Zhou YLZB1613* (CDBI)
*R. pungens* Cambess. var. *oldhamii* (Miq.) Maxim. 1	*R.* subg. *Idaeobatus*	*R.* sect. *Idaeobatus*	2*x*	China: Shaanxi, Taibai, *X. H. Xiong 1332* (CDBI)
*R. pungens* Cambess. var. *oldhamii* (Miq.) Maxim. 2	*R.* subg. *Idaeobatus*	*R.* sect. *Idaeobatus*	2*x*	China: Gansu, Longnan, *X. H. Xiong 1337* (CDBI)
*R. pungens* Cambess. var. *oldhamii* (Miq.) Maxim. 3	*R.* subg. *Idaeobatus*	*R.* sect. *Idaeobatus*	2*x*	China: Sichuan, Baoxing, *X. H. Xiong 2008* (CDBI)
*R. pungens* Cambess. var. *ternatus* Cardot	—	*R.* sect. *Idaeobatus*	NA	China: Chongqing, Chengkou, *X. H. Xiong 1197* (CDBI)
*R. rosifolius* Sm. 1	*R.* subg. *Idaeobatus*	*R.* sect. *Idaeobatus*	2*x*	China: Jiangxi, Ganzhou, *X. H. Xiong 1732* (CDBI)
*R. rosifolius* Sm. 2	*R.* subg. *Idaeobatus*	*R.* sect. *Idaeobatus*	2*x*	China: Zhejiang, Qingyuan, *X. H. Xiong 1913* (CDBI)
*R. rosifolius* Sm. 3	*R.* subg. *Idaeobatus*	*R.* sect. *Idaeobatus*	2*x*	China: Jiangxi, Jinggangshan, *X. H. Xiong 1975* (CDBI)
*R. sikkimensis* Hook. f. 1	*R.* subg. *Idaeobatus*	*R.* sect. *Idaeobatus*	NA	China: Xizang, Motuo, *X. H. Xiong 984* (CDBI)
*R. sikkimensis* Hook. f. 2	*R.* subg. *Idaeobatus*	*R.* sect. *Idaeobatus*	NA	China: Xizang, Motuo, *W. B. Ju et H. N. Deng YLZB0833* (CDBI)
*R. stans* Focke 1	*R.* subg. *Idaeobatus*	*R.* sect. *Idaeobatus*	2*x*	China: Yunnan, Shangri-la, *X. H. Xiong 1996* (CDBI)
*R. stans* Focke 2	*R.* subg. *Idaeobatus*	*R.* sect. *Idaeobatus*	2*x*	China: Sichuan, Jinyang, *X. H. Xiong 2021* (CDBI)
*R. stans* Focke 3	*R.* subg. *Idaeobatus*	*R.* sect. *Idaeobatus*	2*x*	China: Sichuan, Muli, *X. H. Xiong 2031* (CDBI)
*R.* cf. *stans* Focke 1	*R.* subg. *Idaeobatus*	*R.* sect. *Idaeobatus*	2*x*	China: Xizang, Cuona, *X. H. Xiong 1577* (CDBI)
*R.* cf. *stans* Focke 2	*R.* subg. *Idaeobatus*	*R.* sect. *Idaeobatus*	2*x*	China: Xizang, Milin, *X. H. Xiong 1584* (CDBI)
*R. subcoreanus* T. T. Yu et L. T. Lu 1	—	*R.* sect. *Idaeobatus*	NA	China: Shaanxi, Hanzhong, *X. H. Xiong 1327* (CDBI)
*R. subcoreanus* T. T. Yu et L. T. Lu 2	—	*R.* sect. *Idaeobatus*	NA	China: Shaanxi, Hanzhong, *X. H. Xiong 1329* (CDBI)
*R.* cf. *subcoreanus* T. T. Yu et L. T. Lu	—	*R.* sect. *Idaeobatus*	NA	China: Shaanxi, Hanzhong, *X. H. Xiong 1328* (CDBI)
*R. subinopertus* T. T. Yu et L. T. Lu	—	*R.* sect. *Idaeobatus*	2*x*	China: Xizang, Motuo, *X. H. Xiong 1407* (CDBI)
*R. subornatus* Focke 1	*R.* subg. *Idaeobatus*	*R.* sect. *Idaeobatus*	NA	China: Sichuan, Kangding, *X. H. Xiong 1314* (CDBI)
*R. subornatus* Focke 2	*R.* subg. *Idaeobatus*	*R.* sect. *Idaeobatus*	NA	China: Xizang, Linzhi, *X. H. Xiong 1374* (CDBI)
*R. subornatus* Focke 3	*R.* subg. *Idaeobatus*	*R.* sect. *Idaeobatus*	NA	China: Xizang, Milin, *X. H. Xiong 1582* (CDBI)
*R. subornatus* Focke 4	*R.* subg. *Idaeobatus*	*R.* sect. *Idaeobatus*	NA	China: Sichuan, Jinyang, *X. H. Xiong 2028* (CDBI)
*R. subornatus* Focke var. *melanadenus* Focke 1	*R.* subg. *Idaeobatus*	*R.* sect. *Idaeobatus*	NA	China: Sichuan, Kangding, *X. H. Xiong 1264* (CDBI)
*R. subornatus* Focke var. *melanadenus* Focke 2	*R.* subg. *Idaeobatus*	*R.* sect. *Idaeobatus*	NA	China: Sichuan, Baoxing, *X. H. Xiong 2011* (CDBI)
*R. subtibetanus* Hand.-Mazz. 1	—	*R.* sect. *Idaeobatus*	NA	China: Sichuan, *X. H. Xiong 1762* (CDBI)
*R. subtibetanus* Hand.-Mazz. 2	—	*R.* sect. *Idaeobatus*	NA	China: Sichuan, Kangding, *X. H. Xiong 1999* (CDBI)
*R. sumatranus* Miq. 1	*R.* subg. *Malachobatus* (1910, erroneous), *R.* subg. *Idaeobatus* (1911, correct)	*R.* sect. *Idaeobatus*	2*x*	China: Xizang, Motuo, *X. H. Xiong 1392* (CDBI)
*R. sumatranus* Miq. 2	*R.* subg. *Malachobatus* (1910, erroneous), *R.* subg. *Idaeobatus* (1912, correct)	*R.* sect. *Idaeobatus*	2*x*	China: Sichuan, Luzhou, *X. H. Xiong 1805* (CDBI)
*R. sumatranus* Miq. 3	*R.* subg. *Malachobatus* (1910, erroneous), *R.* subg. *Idaeobatus* (1913, correct)	*R.* sect. *Idaeobatus*	2*x*	China: Jiangxi, Jinggangshan, *X. H. Xiong 1989* (CDBI)
*R. thibetanus* Franch. 1	*R.* subg. *Idaeobatus*	*R.* sect. *Idaeobatus*	2*x*	China: Gansu, Longnan, *X. H. Xiong 1336* (CDBI)
*R. thibetanus* Franch. 2	*R.* subg. *Idaeobatus*	*R.* sect. *Idaeobatus*	2*x*	China: Sichuan, Chengdu, *X. H. Xiong 1529* (CDBI)
*R. thibetanus* Franch. 3	*R.* subg. *Idaeobatus*	*R.* sect. *Idaeobatus*	2*x*	China: Sichuan, *X. H. Xiong 1764* (CDBI)
*R. trijugus* Focke 1	*R.* subg. *Idaeobatus*	*R.* sect. *Idaeobatus*	NA	China: Sichuan, Danba, *X. H. Xiong 2167* (CDBI)
*R. trijugus* Focke 2	*R.* subg. *Idaeobatus*	*R.* sect. *Idaeobatus*	NA	China: Sichuan, Jinchuan, *X. H. Xiong 2175* (CDBI)
*R.* cf. *tsangii* Merr.	—	*R.* sect. *Idaeobatus*	2*x*	China: Sichuan, *X. H. Xiong 1783* (CDBI)
*R. wallichianus* Wight et Arn. 1 (*R. pinfaensis* H. Lév. et Vaniot)	*R.* subg. *Idaeobatus*	*R.* sect. *Idaeobatus*	2*x*	China: Yunnan, *X. H. Xiong 1743* (CDBI)
*R. wallichianus* Wight et Arn. 2	*R.* subg. *Idaeobatus*	*R.* sect. *Idaeobatus*	2*x*	China: Sichuan, Chengdu, *X. H. Xiong 1785* (CDBI)
*R. wallichianus* Wight et Arn. 3	*R.* subg. *Idaeobatus*	*R.* sect. *Idaeobatus*	2*x*	China: Sichuan, Luzhou, *X. H. Xiong 1799* (CDBI)
*R. wallichianus* Wight et Arn. 4	*R.* subg. *Idaeobatus*	*R.* sect. *Idaeobatus*	2*x*	China: Guizhou, *X. H. Xiong 1820* (CDBI)
*R. wushanensis* T. T. Yu et L. T. Lu	—	*R.* sect. *Idaeobatus*	NA	China: Chongqing, Wushan, *X. H. Xiong 1183* (CDBI)
*R. paniculatus* Sm. 1	*R.* subg. *Malachobatus*	*R.* sect. *Malachobatus*	2*x*	China: Xizang, Motuo, *X. H. Xiong 966* (CDBI)
*R. paniculatus* Sm. 2	*R.* subg. *Malachobatus*	*R.* sect. *Malachobatus*	2*x*	China: Xizang, Motuo, *X. H. Xiong 969* (CDBI)
*R. paniculatus* Sm. 3	*R.* subg. *Malachobatus*	*R.* sect. *Malachobatus*	2*x*	China: Xizang, Motuo, *X. H. Xiong 971* (CDBI)
*R. allegheniensis* Porter	*R.* subg. *Rubus*	—	2*x*, 3*x*, 4*x*	USA: Pennsylvania, Lehighton, *T. A. Merkel s. n.* (NPGS, Accession PI 553970)
*R. argutus* Link	*R.* subg. *Rubus*	—	2*x*, 3*x*	USA: South Carolina, *J. R. Ballington et J. A. Payne s. n.* (NPGS, Accession PI 606490)
*R. caesius* L. 1	*R.* subg. *Rubus*	*R.* sect. *Rubus*	4*x*	Russia: Stavropol, *H. J. Brooks s. n.* (NPGS, Accession PI 324058)
*R. caesius* L. 2	*R.* subg. *Rubus*	*R.* sect. *Rubus*	4*x*	Spain: Zaragoza, *Erickson s. n.* (NPGS, Accession PI 420407)
*R. canadensis* L.	*R.* subg. *Rubus*	—	2*x*, 3*x*	USA: Georgia, *J. R. Ballington s. n.* (NPGS, Accession PI 553141)
*R. canescens* DC.	*R.* subg. *Rubus*	—	2*x*	Former Serbia and Montenegro: *L. Aladzajkov s. n.* (NPGS, Accession PI 379537)
*R. hispidus* L. 1	*R.* subg. *Rubus*	—	2*x*	USA: North Carolina, Beaufort County, *J. R. Ballington s. n.* (NPGS, Accession PI 606507)
*R. hispidus* L. 2	*R.* subg. *Rubus*	—	2*x*	USA: Tennessee, Cumberland County, *Ano. s. n.* (NPGS, Accession PI 618281)
*R. sanctus* Schreb. 1	*R.* subg. *Rubus*	—	2*x*	Pakistan: Chitral District, *D. Brenner et M. Thompson s. n.* (NPGS, Accession PI 553879)
*R. sanctus* Schreb. 2	*R.* subg. *Rubus*	—	2*x*	Syria: Damascus, *M. Thompson s. n.* (NPGS, Accession PI 553930)
*R. trivialis* Michx.	*R.* subg. *Rubus*	—	2*x*	USA: Texas, Austin, *L. Whiting s. n.* (NPGS, Accession PI 553704)
*R. ulmifolius* Schott 1	*R.* subg. *Rubus*	—	2*x*	Syria: Damascus, *M. Thompson s. n.* (NPGS, Accession PI 553931)
*R. ulmifolius* Schott 2	*R.* subg. *Rubus*	—	2*x*	USA: California, Napa County, *L. Clark s. n.* (NPGS, Accession PI 672650)
Outgroups				
*Fragaria daltoniana* J. Gay			2*x*	China: Xizang, Milin, *X. H. Xiong 1590* (CDBI)
*Rosa cymosa* Tratt. 1			2*x*	China: Jiangxi, Dayu, *X. H. Xiong 803-2* (CDBI)
*R. cymosa* Tratt. 2			2*x*	China: Jiangxi, Xingan, *X. H. Xiong 813-1* (CDBI)
*R. luciae* Franch. et Rochebr.			2*x*	China: Zhejiang, Wenzhou, *C. Zhang s. n.* (CDBI)

Notes: Ploidy is mainly from Thompson [13] and the Chromosome Counts Database (CCDB) [87]. NA indicates data not available. Herbarium acronyms follow Index Herbariorum [88]. NPGS—U.S. National Plant Germplasm System.
